# Financial barriers and adverse clinical outcomes among patients with cardiovascular-related chronic diseases: a cohort study

**DOI:** 10.1186/s12916-017-0788-6

**Published:** 2017-02-15

**Authors:** David J. T. Campbell, Braden J. Manns, Robert G. Weaver, Brenda R. Hemmelgarn, Kathryn M. King-Shier, Claudia Sanmartin

**Affiliations:** 1 Department of Medicine, Cumming School of Medicine, University of Calgary, Health Sciences Centre, Room G236, 3330 Hospital Dr NW, Calgary, AB T2N 4N1 Canada; 2 Department of Community Health Sciences, Cumming School of Medicine, University of Calgary, Calgary, AB Canada; 3 O’Brien Institute for Public Health, University of Calgary, Calgary, AB Canada; 4 Libin Cardiovascular Institute of Alberta, University of Calgary, Calgary, AB Canada; 5 Faculty of Nursing, University of Calgary, Calgary, AB Canada; 6 Health Analysis Division, Statistics Canada, Ottawa, ON Canada

## Abstract

**Background:**

Some patients with cardiovascular-related chronic diseases such as diabetes and heart disease report financial barriers to achieving optimal health. Previous surveys report that the perception of having a financial barrier is associated with self-reported adverse clinical outcomes. We sought to confirm these findings using linked survey and administrative data to determine, among patients with cardiovascular-related chronic diseases, if there is an association between perceived financial barriers and the outcomes of: (1) disease-related hospitalizations, (2) all-cause mortality and (3) inpatient healthcare costs.

**Methods:**

We used ten cycles of the nationally representative Canadian Community Health Survey (administered between 2000 and 2011) to identify a cohort of adults aged 45 and older with hypertension, diabetes, heart disease or stroke. Perceived financial barriers to various aspects of chronic disease care and self-management were identified (including medications, healthful food and home care) from the survey questions, using similar questions to those used in previous studies. The cohort was linked to administrative data sources for outcome ascertainment (Discharge Abstract Database, Canadian Mortality Database, Patient Cost Estimator). We utilized Poisson regression techniques, adjusting for potential confounding variables (age, sex, education, multimorbidity, smoking status), to assess for associations between perceived financial barriers and disease-related hospitalization and all-cause mortality. We used gross costing methodology and a variety of modelling approaches to assess the impact of financial barriers on hospital costs.

**Results:**

We identified a cohort of 120,752 individuals over the age of 45 years with one or more of the following: hypertension, diabetes, heart disease or stroke. One in ten experienced financial barriers to at least one aspect of their care, with the two most common being financial barriers to accessing medications and healthful food. Even after adjustment, those with at least one financial barrier had an increased rate of disease-related hospitalization and mortality compared to those without financial barriers with adjusted incidence rate ratios of 1.36 (95% CI: 1.29–1.44) and 1.24 (1.16–1.32), respectively. Furthermore, having a financial barrier to care was associated with 30% higher inpatient costs compared to those without financial barriers.

**Discussion:**

This study, using novel linked national survey and administrative data, demonstrates that chronic disease patients with perceived financial barriers have worse outcomes and higher resource utilization, corroborating the findings from prior self-report studies. The overall exposure remained associated with the primary outcome even in spite of adjustment for income. This suggests that a patient’s perception of a financial barrier might be used in clinical and research settings as an additional measure along with standard measures of socioeconomic status (ie. income, education, social status).

**Conclusions:**

After adjusting for relevant covariates, perceiving a financial barrier was associated with increased rates of hospitalization and mortality and higher hospital costs compared to those without financial barriers. The demonstrable association with adverse outcomes and increased costs seen in this study may provide an impetus for policymakers to seek to invest in interventions which minimize the impact of financial barriers.

## Background

As the populace in Western countries continues to age, the prevalence of chronic diseases is also on the rise, with three-quarters of seniors reporting at least one chronic disease [1]. Cardiovascular-related chronic diseases are the leading cause of hospitalization [2] and also the predominant cause of premature death and disability [3].

Patients in countries around the world experience financial barriers to the care they require to manage their chronic conditions [4]. In Canada, 10–12% of patients with chronic diseases face financial barriers [5, 6]. This happens in spite of Canada’s single payer healthcare system. Canadian provinces' public health insurance provides universal full coverage for physician and hospital services, but coverage for outpatient services, including medications, is inconsistent and has been described as a ’patchwork’ across Canada’s provinces, and even within provinces across different age and sociodemographic groups [7]. Canadians with cardiovascular-related chronic diseases who perceived financial barriers self-reported being 70% more likely to require emergency department visits and/or hospitalizations for their chronic diseases [5]. Similar results have been reported in the USA, where Americans with financial barriers were more likely to report having a cardiac-related readmission to hospital following an initial myocardial infarction [8]. These prior studies are based on self-reported outcomes and may be prone to bias, as patients may not be able to accurately identify if their hospitalization was in fact related to their chronic disease. Only one previous small study examined the relationship between perceived financial barriers and an objectively measured outcome: recurrent cardiac events [9].

We hypothesized that patients with chronic disease who experience financial barriers would have more hospitalizations and a higher mortality rate and would accrue higher healthcare costs than those without financial barriers. To overcome the limitations of prior studies in this area, we linked national survey data with administrative health data to determine the association between perceived financial barriers and objectively documented disease-related hospitalizations (primary outcome) as well as all-cause mortality and costs associated with disease-related hospitalizations (secondary outcomes).

## Methods

### Study context

Canada is a federation with considerable autonomy vested in individual provinces and territories. The delivery of health and healthcare insurance falls under the purview of provincial and territorial governments. Despite significant inter-provincial differences, Canada has had universal publicly funded insurance for hospital and physician services since 1966 and 1972, respectively. Under the Canada Health Act (1982), Canadian citizens and residents have full access to these services without being compelled to pay point-of-care charges [10].

Public insurance plans for other services, such as medications and allied healthcare, are not provided universally and differ between provinces [7]. Those who do qualify for public supplemental health insurance are often still left to contribute significantly to healthcare expenditures through copayments and deductibles [11].

Within this context, Canadians may encounter a variety of financial barriers to accessing care for their chronic conditions. Patients may face direct costs for non-insured services including medications, allied healthcare provider fees and home care. Patients may also face indirect costs associated with accessing services which are fully insured. For example, the costs that patients are required to pay for transportation, parking and childcare, as well as lost income from time away from work, may all be disincentives to attending physician appointments or completing laboratory investigations.

### Data sources

The data source for this project is a novel dataset which linked the 2000 to 2011 Canadian Community Health Survey (CCHS) linked to the Discharge Abstract Database (1996–2013) and Canadian Mortality Database (2000–2011). The linkage was conducted by Statistics Canada using probabilistic methods based on common variables including date of birth, postal codes, sex, health insurance number and name [12]. The linkage was conducted among CCHS respondents who agreed to link and share their information. Additional postal code information derived from tax filer information was used to augment the linkage by accounting for respondent moves over time [13].

The CCHS is a national cross-sectional survey that has been conducted annually since 2000. The survey is administered by Statistics Canada and collects information on the health, health behaviours and healthcare use of the non-institutionalized population aged 12 years and older. The survey excludes full-time members of the Canadian Forces and residents of reserves and some remote areas, together representing about 4% of the target population. The CCHS was first conducted in 2000/2001 (cycle 1) and again in 2003 (cycle 2) and 2005 (cycle 3), each time with a sample size of approximately 130,000. Starting in 2007, the survey was conducted annually (sample size of 65,000). Response rates ranged from 69.8% to 78.9% [14]. CCHS respondents who provided their consent to share and link their survey responses were eligible for linkage.

We also used responses to health surveys that have been administered to subsamples of CCHS respondents to obtain greater detail on a variety of topics. These included the 2007 Rapid Response module about prescription drug expenditures (*n* = 10,500); the Survey on Living with Chronic Diseases in Canada from 2009 - hypertension (*n* = 6338) and 2011 - diabetes (*n* = 3747); and the 2012 Barriers to Care for Persons with Chronic Health Conditions survey administered to respondents with chronic conditions living in four western provinces (*n* = 1849).

The Discharge Abstract Database captures administrative and clinical data for all patients discharged from acute care hospitals in Canada (excluding Quebec) [15]. The data are coded by trained hospital coders and transmitted to provincial/territorial ministries of health, who forward it to the Canadian Institute for Health Information [15]. One most responsible diagnosis along with up to 24 secondary diagnoses are coded according to the International Classification of Disease framework [16]. Hospital records were available from 1 April 1996 to 31 March 2013. For each individual, the pertinent hospital information extracted includes (1) number of hospitalizations in the follow-up period; (2) the most responsible diagnosis documented for each hospitalization; (3) coronary revascularization procedures (percutaneous coronary intervention and coronary artery bypass grafting); (4) length of stay of each acute care hospitalization; and (5) gross costing information assigned to each hospitalization to permit linkage to costing data.

The Canadian Mortality Database collects "information annually from all provincial and territorial vital statistics registries on all deaths in Canada” [17]. Mortality data were available from 1 January 2000 to 31 December 2011.

In Canada, each hospital encounter is assigned to a Major Clinical Category (similar to a diagnosis-related grouping) and a more granular Case-Mix Grouper (CMG), which is a code assigned to each hospitalization based on intensity of resources required during that stay [18]. Since 2009, the Patient Cost Estimator, generated by the Canadian Institute for Health Information, provides annualized tables of estimated mean costs associated with each CMG code [19]. Linkage via CMG code allows for an estimation of costs associated with each inpatient encounter.

### Study design and cohort creation

**Fig. 1:**
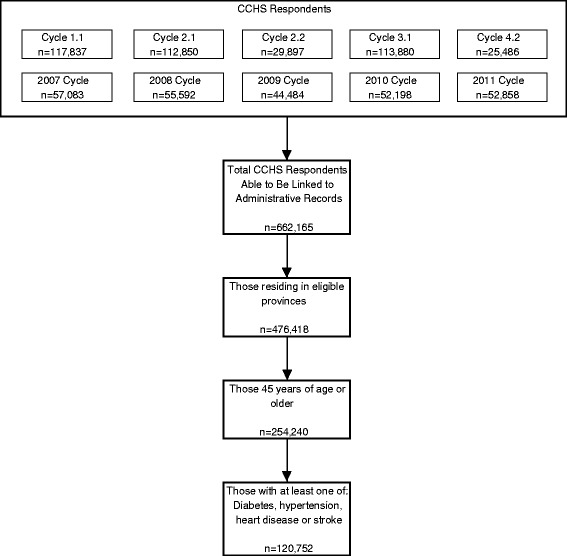
Cohort selection

We used an observational cohort design. Our cohort was defined by all CCHS respondents eligible for linkage who were at least 45 years old at the time of survey administration, had self-reported having at least one of the chronic conditions of interest (heart disease, diabetes, stroke, hypertension) and were residents of one of the provinces that reported hospitalization data consistently throughout the entire follow-up period (i.e. all except Manitoba, Quebec and the territories) (see Fig. 1).

### Exposure definition

The exposure of interest was perceiving a financial or cost-related barrier to care as defined by responses to the health surveys. In our preceding qualitative study [20, 21], we identified that patients may experience financial barriers to a variety of goods and services that are required for disease self-management. These include (1) medications (given that cost sharing is often required); (2) indirect costs related to use of covered healthcare provider visits and laboratory investigations as well as direct costs related to healthcare provider visits that are not universally covered (eye exams and dental care); (3) access to healthful food; (4) ability to make health behaviour modifications (physical activity, weight loss and smoking cessation); and (5) home care (only those ≥75 years of age were included for this barrier). Finally, within several cycles of the CCHS, individuals were also asked whether they had an unmet need for healthcare due to cost.

The survey questions used to define these exposures are given in Appendix 1. Given that not all CCHS respondents were asked about all types of barriers, these were each analysed as individual cohorts. We conducted a final analysis by combining all eligible individuals who noted at least one of the above financial barriers.

### Outcome definition

The primary outcome was disease-related hospitalization, defined as a stay of longer than 1 day in a Canadian acute care facility for which the most responsible diagnosis was coded as either a cardiovascular or diabetes-related cause, or during which the patient underwent coronary revascularization, defined using administrative data codes (Appendix 2). The diagnosis coded as most responsible in the Discharge Abstract Database is that which was responsible for the greatest portion of the length of stay. In previous validation studies, this diagnosis has been more reliably coded than the other diagnoses, which often represent comorbid conditions [22]. Hospitalizations with a duration less than 1 day were excluded, as these are generally hospital day surgeries and procedures which are (1) less reliably captured in the administrative data; (2) less likely to have pertinence to cardiovascular disease or diabetes and (3) more likely to represent a planned procedure (such as an elective diagnostic angiogram) than one representing a true event of interest. Furthermore, emergency department visits are not represented within the Discharge Abstract Database.

The secondary outcomes of interest were all-cause mortality — defined from the Canadian Mortality Database — and inpatient healthcare costs for disease-related hospitalizations. Since validated hospital costing data were only available for those admitted over a 3-year period (2010–2012), we first established a mean cost per hospital-day for disease-related hospitalizations by dividing the sum of the estimated costs of all disease-related hospitalizations during this period by the total length of hospital stay. We combined average cost per day with length of stay data for the entire cohort to yield an estimate of the costs associated with each disease-related hospitalization in the entire dataset.

### Covariates

Based on prior work, we identified a number of covariates that have been shown to be independently associated with hospitalizations for chronic conditions and are therefore important to consider as potential confounders [23]. These included age, sex, smoking status, comorbidities and socioeconomic status. Age, education, smoking status and multimorbidity were included as categorical variables, as defined in Table 1. We also included mental health comorbidity, defined as anyone self-reporting a prior diagnosis of mood or anxiety disorders. Socioeconomic status was represented by level of educational attainment, as education has been shown to be among the most representative indicators of overall socioeconomic status [24]. Furthermore, income was found to be very highly collinear with financial barriers, so education was chosen as the marker of socioeconomic status. Finally, we assessed for effect modification by age using an interaction term (age category * financial barrier) and the corresponding Wald tests.

**Table 1:** Participant characteristics

		Overall (*n* = 120,752)		Any financial barrier (*n* = 12,303)﻿*		No financial barrier (*n* = 108,449)		*p* (chi square)
		*n*	%	*n*	%	*n*	%	
Age	45–64 years	50,228	41.6	7470	60.7	42,758	39.4	<0.001
	65–74 years	33,951	28.1	2766	22.5	31,185	28.8	
	75+	36,573	30.3	2067	16.8	34,506	31.8	
Demographic characteristics	Sex: male	53,098	44.0	4882	39.7	48,216	44.5	<0.001
	Marital Status: married	67,725	56.1	5010	40.8	62,715	57.9	<0.001
	Residence: rural	34,386	28.5	3320	27.0	31,066	28.7	<0.001
Ethnicity	White	111,639	92.8	10,690	87.4	100,949	93.5	<0.001
	Indigenous	3030	2.5	717	5.9	2313	2.1	
	Other	5585	4.6	831	6.8	4754	4.4	
	Not stated	498		65		433		
Household income ($ CAD)	0–29,999	44,638	43.9	8108	72.2	36,530	40.4	<0.001
	30,000–49,999	25,802	25.4	1942	17.3	23,860	26.4	
	50,000–69,999	18,630	18.3	818	7.3	17,812	19.7	
	70,000 +	12,628	12.4	367	3.2	12,261	13.5	
	Not stated	19,054		1068		17,986		
Education	Less than secondary	42,199	35.3	5248	43.0	36,951	34.4	<0.001
	Secondary graduate	26,026	21.8	2545	20.9	23,481	21.9	
	Post-secondary graduate	51,269	42.9	4398	36.1	46,871	43.7	
	Not stated	1258		112		1146		
Smoking status	Current smoker	49,309	40.8	4157	33.8	45,152	41.6	<0.001
	Former smoker	52,395	43.4	4423	36.0	47,972	44.2	
	Never smoker	19,048	15.8	3723	30.2	15,325	14.1	
Type of condition	Hypertension	99,611	82.5	9962	81.0	89,649	82.7	<0.001
	Diabetes	30,055	24.9	3821	31.1	26,234	24.2	<0.001
	Heart disease	32,319	26.8	3665	29.8	28,654	26.4	<0.001
	Stroke	6976	5.8	1054	8.6	5922	5.5	<0.001
	Multimorbidity†﻿	39,612	32.8	4852	39.4	34,760	32.1	<0.001
	Mental illness‡	12,215	12.0	2787	28.2	9428	10.3	<0.001
BMI class (corrected for self-report [35])	Obese	39,401	37.7	4904	45.7	34,497	39,401	<0.001
	Overweight	40,210	38.4	3468	32.3	36,742	40,210	
	Normal/underweight	24,991	23.9	2371	22.1	22,620	24,991	
	Not stated	16,150		1560		14,590	16,150	
Self-perceived health	Excellent/very good	40,378	33.5	2194	17.9	38,184	35.3	<0.001
	Good	42,725	35.4	3664	29.9	39,061	36.1	
	Fair/poor	37,453	31.1	6416	52.2	31,037	28.7	
	Not stated	196.0		29.0		167		

*Those who identified having at least one type of financial barrier to care - see Appendix

†Multimorbidity denotes those who have more than one of heart disease, diabetes, hypertension and/or stroke

‡Mental illness denotes those who self-reported having either anxiety or mood disorders

### Statistical analysis

As the follow-up time could vary for each participant (based on dates of cohort entry and death), we calculated rates of events to take into account differing observation times. We defined the index date as the first day of administration of the cycle of the survey to which the participant responded (for example 1 January 2010 for anyone completing the 2010 cycle). Since the perceived financial barrier and the chronic disease of interest were unlikely to be new at the time of the survey, we assessed the primary outcome over 5 years: 2 years prior to the index date, as well as 3 years prospectively. For the mortality outcome, follow-up was from the time of survey administration to the date of death or end of follow-up for mortality data (31 December 2011).

For the hospitalization outcome (count data), we initially fit Poisson regression models. We tested for overdispersion using the likelihood ratio test and used negative binomial models in such cases [25]. For the mortality outcome, we used modified Poisson regression models with robust standard errors [26] to generate mortality rate ratios. We present results for unadjusted, fully adjusted and final reduced models after conducting backwards elimination procedures: covariates were sequentially removed from the model one at a time, and those covariates whose removal changed the point estimate for financial barriers by >10% were considered true confounders and were retained in final models (other than age and sex which, by default, were retained in models). We back- calculated adjusted rates from the reduced models by adjusting to the overall means/proportions of the covariates.

For the costing analysis, we fit various models, given the well-documented difficulty in analysing skewed cost data [27]. We started with ordinary least squares (OLS) linear regression, but also considered other generalized linear models (GLMs). We used several GLM models and used the modified Park test [28] to determine which GLM distribution provided the best fit for our data. According to the assessment of the fit of these various models, we found that the GLM with a Poisson distribution and log link was most appropriate.

Cases with missing data were left as missing in analyses; no imputation of data was undertaken. People with missing exposure statuses who were not asked the pertinent questions were not considered exposed or unexposed but were excluded from the analysis altogether. All analyses were conducted with Stata v.11.0 (Stata, College Station, TX, USA). Ethics approval was received from the University of Calgary’s Conjoint Health Research Ethics Board, and all procedures were followed in accordance with the ethics board and Statistics Canada.

## Results

From the initial 751,189 CCHS respondents, we identified 120,752 individuals who met all study inclusion criteria (Fig. 1). The total follow-up time for the hospitalization outcome was 586,900 patient years (average: 4.86 years/participant), and that for the mortality outcome was 573,200 patient years (average: 4.75 years/participant).

Overall, study participants were predominantly white, married, urban-dwelling and female (Table 1). Participants with a financial barrier were considerably different from those with no financial barrier across all clinical and sociodemographic characteristics and were more likely to be younger, unmarried females with low income, lower education, multimorbidity and worse self-perceived health.

The barriers most commonly cited were financial barriers to accessing healthful food (8.9% of those asked these questions) and medications (7.5% of those asked) (Table 2). Overall, 10.2% of respondents perceived a financial barrier to at least one aspect of their chronic disease management, though this is likely an underestimate, as not all respondents were asked about financial barriers to all relevant aspects of self-management.

**Table 2:** Rate of outcomes and incidence rate ratios, by financial barrier type

	Outcome	Adjusted incidence rate^a^ (95% CI) (no. outcomes/year/1000 population)		Incidence rate ratio (95% CI)			
		No financial barrier	Financial barrier	Crude	Fully adjusted	Reduced final	Final covariates
Home care (only >75 years)	*N* (%)	23,397 (98.8)	280 (1.2)				
	DR-Hosp	60.2 (58.3-62.2)	80.6 (59.8-101.5)	1.43 (1.10-1.88)	1.36 (1.05-1.76)	1.34 (1.03-1.74)	Sex, MM
	Mortality	59.6 (58.1-61.2)	76.1 (60.5-91.7)	1.26 (1.05-1.51)	1.15 (0.93-1.42)	1.28 (1.04-1.57)	Sex
Medications	*N* (%)	2782 (92.5)	224 (7.5)				
	DR-Hosp	26.0 (22.6-29.3)	40.2 (24.4-56.1)	1.24 (0.82-1.88)	1.39 (0.92-2.12)	1.55 (1.02-2.35)	Age, sex
	Mortality	14.2 (11.0-17.3)	6.7 (0.1-13.3)	0.40 (0.15-1.07)	0.39 (0.14-1.07)	0.47 (0.18-1.27)	Age, sex
Healthcare provider/test	*N* (%)	101,040 (97.7)	2383 (2.3)				
	DR-Hosp	35.0 (34.3-35.7)	43.3 (37.8-48.7)	0.98 (0.86-1.11)	1.08 (0.95-1.24)	1.24 (1.09-1.40)	Age, sex
	Mortality	20.8 (20.3-21.3)	25.3 (21.9-28.7)	0.83 (0.73-0.95)	1.09 (0.93-1.26)	1.22 (1.06-1.39)	Age, sex
Healthful food	*N* (%)	97,754 (91.1)	9506 (8.9)				
	DR-Hosp	35.5 (34.7-36.2)	59.5 (56.0-63.0)	1.25 (1.17-1.33)	1.31 (1.22-1.41)	1.68 (1.56-1.79)	Age, sex, MM
	Mortality	6.6 (6.4-6.8)	9.3 (8.6-9.9)	1.08 (1.02-1.14)	1.32 (1.23-1.42)	1.41 (1.31-1.51)	Age, sex, MH, SM
Health behaviour modification	*N* (%)	18,142 (98.4)	289 (1.6)				
	DR-Hosp	35.4 (33.8-37.0)	37.9 (24.1-51.6)	0.84 (0.58-1.22)	1.11 (0.77-1.60)	1.07 (0.74-1.54)	Age, sex
	Mortality	18.0 (16.5-19.5)	15.5 (5.9-25.1)	0.56 (0.30-1.02)	0.89 (0.49-1.62)	0.90 (0.49-1.63)	Age, sex
Unmet need due to cost	*N* (%)	70,826 (99.0)	750 (1.0)				
	DR-Hosp	34.6 (33.8-35.5)	44.1 (35.0-53.1)	1.15 (0.93-1.43)	1.24 (0.97-1.58)	1.27 (1.03-1.56)	Age, sex, MM
	Mortality	23.2 (22.6-23.7)	21.1 (16.6-25.6)	0.69 (0.56-0.83)	0.83 (0.64-1.06)	0.90 (0.74-1.09)	Age, sex
At least 1 financial barrier	*N* (%)	110,123 (91.2)	12,303 (10.2)				
	DR-Hosp	31.7 (31.1-32.3)	43.1 (40.9-45.3)	1.19 (1.13-1.27)	1.23 (1.16-1.31)	1.36 (1.29-1.44)	Age, sex, MM
	Mortality	7.7 (7.5-7.9)	9.6 (9.0-10.1)	1.02 (0.97-1.07)	1.09 (1.03-1.16)	1.24 (1.16-1.32)	Age, sex, MH

*DR-Hosp* disease-related hospitalization

*MH* mental health comorbidity

*MM* multimorbidity

*SM* smoking

^a^Adjusted using the reduced models to the overall means/proportions of covariates

**Fig. 2:**
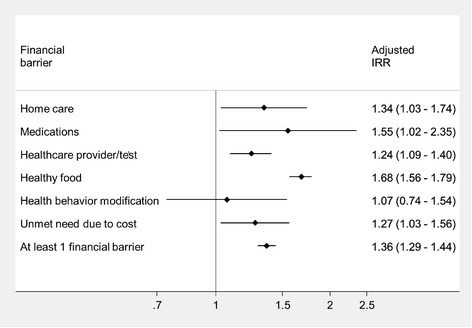
Forest plot of disease-related hospitalization

We found that after adjustment and model reduction, there was a significant positive association between experiencing financial barriers and both clinical outcomes of interest (Table 2). Those with at least one financial barrier had a 36% higher rate of hospitalization for disease-related causes than those without financial barriers (incidence rate ratio (IRR) 1.36, 95% CI 1.29–1.44). With the exception of financial barriers to health behavior modification, the individual financial barriers were all significantly associated with disease-related hospitalizations with IRRs ranging from 1.24 (financial barriers to seeing healthcare providers or having tests) to 1.68 (financial barrier to eating healthful food) (Fig. 2). Despite concern regarding collinearity and overmodelling with the inclusion of income in the models, we conducted a sensitivity analysis by adding income to the final reduced models (Table 3). This demonstrated that some barriers, including the aggregated exposure, were attenuated but remained significantly associated with the primary outcome even adjusting for income (IRR 1.16, 95% CI 1.09–1.23).

**Table 3:** Sensitivity analysis of primary outcome, with income added to final reduced models

Financial barrier	IRR (95% CI)
Home care	1.33 (1.01–1.77)
Medications	1.20 (0.76–1.92)
MD/test	1.05 (0.92–1.21)
Healthful food	1.34 (1.25–1.43)
Lifestyle	0.99 (0.67–1.45)
Unmet need	1.11 (0.90–1.37)
At least 1 financial barrier	1.16 (1.09–1.23)

Those with any financial barrier had an adjusted mortality rate that was 24% higher than those without financial barriers (IRR 1.24, 95% CI 1.16–1.32). The individual barriers that were significantly associated with mortality included financial barriers to homecare, healthcare providers/tests and healthful food.

The costing analysis, using OLS linear regression, demonstrated that on average those with financial barriers incurred inpatient disease-related healthcare costs of $1724 over a 5-year period, compared to those without financial barriers ($1360). Having a financial barrier was associated with a higher cost of $364/patient over the 5-year observation period (95% CI: $204–524). We contrasted this finding with the ideal model, the GLM with Poisson distribution and log link, which demonstrated that those with financial barriers had 37% higher inpatient costs — very similar to the estimate produced using OLS linear regression.

## Discussion

Using a novel national dataset comprising linked administrative and survey data for adults with cardiovascular-related chronic diseases, we found that the perception of a financial barrier to care was associated with a 36% higher rate of cardiovascular- or diabetes-related hospitalization and a 24% higher mortality rate. This is the first time that data sources with objective outcomes have been used to determine that chronic disease patients with perceived financial barriers have worse outcomes and higher resource utilization, as previous studies have relied upon self-reported outcomes.

The fact that our overall exposure (at least one financial barrier) retained a significant positive association with disease-related hospitalization in spite of adjustment for income suggests that financial barriers may be relevant, at least for some aspects of chronic disease care, regardless of income level. Consistent with prior qualitative research [21], financial barriers appear relevant even for some patients with higher income and may not be experienced universally by those with lower income. This suggests that a patient’s perception of a financial barrier might be used in clinical and research settings as an additional measure along with standard measures of socioeconomic status (i.e. income, education, social status).

Previous studies using the CCHS have estimated that the prevalence of these cardiovascular-related conditions in the Canadian adult population are as follows: 1.2% stroke, 5.0% diabetes, 5.4% heart disease and 15.5% hypertension [29]. Accounting for co-occurrence of these conditions, it is likely that one in five Canadian adults is affected by at least one of these conditions (i.e. 5.6 million people) [30]. We have also shown that of those with these chronic conditions, 10% perceive financial barriers (i.e. 560,000 people) [5]. Since having a financial barrier was associated with an excess hospital cost of $364 per person, Canadian hospital costs may be higher by $200 million dollars over 5 years for those who experience financial barriers. Of note, this only includes the excess costs associated with disease-related hospitalizations, which account for only approximately 20% of all hospitalizations; therefore, the total excess costs may be significantly higher than this estimate.

This study corroborates the findings from the previously conducted self-report studies: perceived financial barriers are associated with a higher likelihood of requiring hospital care for chronic conditions [5, 8]. The observed associations for those with financial barriers is potentially mediated by cost-related non-adherence to medical therapies [5, 6] and health behaviour modification [31], which result in poorer control of chronic diseases and ultimately culminate in hospitalizations and death. We recently completed a grounded theory study on this topic where we found that the impact of financial barriers on patients’ lives is determined by a complex set of individual patient and system factors [21]. The complexity of perceiving financial barriers as well as the demonstrable association with adverse outcomes and increased costs seen in this study may provide an impetus for policymakers to seek to invest in interventions which minimize the impact of financial barriers, such as copayment elimination [32], patient navigation [33] and patient self-advocacy education [34] — though the ultimate impact of these interventions remains inconclusive.

There were some unexpected discrepant findings. Specifically, some financial barriers had a (non-significant) trend towards being protective against mortality. This may reflect that those more likely to perceive an unmet need or a barrier to health behavior modification may be those who are more engaged in their care, or have higher degrees of patient activation and/or health literacy — which may be the factor that confers the lower risk of mortality. This assertion is supported by a previous study that found no association between perceived unmet needs and adverse outcomes [29], suggesting that there may be an offsetting of risk between those who truly have an unmet need and those who may simply be more sensitive to perceiving an unmet need because they are more active participants in their care.

There are several strengths of this study, including the use of a novel dataset linking national surveys containing detailed information on a variety of data not collected routinely and high-quality health administrative data containing objective information on hospital admissions and resource use. There are also limitations of this work. Firstly, as this is an observational study, there is potential for residual confounding (e.g. we had no appropriate measure of disease severity), although we were able to adjust for many of the most important confounders in our regression models. Secondly, some types of financial barriers were only asked of a subset of the sample (e.g. financial barriers to medications), making these analyses underpowered to detect significant associations with the less common outcome of death. Due to the high prevalence of financial barriers to obtaining healthful food and the large proportion of respondents who were asked this question, the associations between having any financial barrier and the outcomes are likely being driven principally by this barrier. However, it is reassuring that when the other types of financial barriers are studied in isolation from each other, consistent associations are found, though some are underpowered. While the outcomes data were longitudinal, the exposure was determined cross-sectionally in the survey, which does not allow for an assessment of how enduring financial barriers may be. Due to inconsistent reporting procedures, we were unable to include data from residents of several jurisdictions (five provinces and territories). Finally, as this study only included Canadian patients, these findings apply specifically to Canadian patients with chronic disease. A number of chronic disease patients in other Western nations report facing financial barriers to various aspects of chronic disease care [4]. Since the USA has an even less comprehensive medication insurance program than Canada, our findings likely apply in the US context.

## Conclusions

Using a novel dataset linking detailed national surveys with high-quality health administrative data, we found that after adjustment, perceiving a financial barrier was associated with higher rates of both disease-related hospitalization and mortality for patients with chronic disease, though unmeasured differences across groups are still possible. We hope that the significance of financial barriers to care as demonstrated by this study will prompt those who collect routine health-related information to incorporate questions about perceived financial barriers more regularly in health surveys in order to enable further analyses on this important topic. These results may be used by health services researchers to inform the design of interventions to address these financial barriers and thus to ultimately minimize the prevalence or mitigate the impact of financial barriers on important outcomes for both patients and healthcare systems.

## Appendix 1

Table 4 describes the survey questions used to define the exposures of interest.

**Table 4:** Definition of exposure (financial barriers)

Healthcare provider or test	ACC_12	In the past 12 months, did you ever experience any difficulties getting the specialist care you needed for a diagnosis or consultation? (Reason: Cost)	CCHS
	ACC_53	In the past 12 months, did you experience difficulties getting routine care that you or a family member required during « regular » office hours (that is, 9:00 am to 5:00 pm, Monday to Friday)? (Reason: Cost)	CCHS
	ACC_55	In the past 12 months, did you experience difficulties getting routine care that you or a family member required during evenings and weekends (that is, 5:00 to 9:00 pm Monday to Friday, or 9:00 am to 5:00 pm, Saturdays and Sundays)? (Reason: Cost)	CCHS
	ACC_63	In the past 12 months, did you ever experience any difficulties getting the immediate care needed for a minor health problem for yourself or a family member that you required during « regular » office hours (that is, 9:00 am to 5:00 pm, Monday to Friday)? (Reason: Cost)	CCHS
	ACC_65	In the past 12 months, did you ever experience any difficulties getting the immediate care needed for a minor health problem for yourself or a family member that you required evenings and weekends (that is, 5:00 to 9:00 pm Monday to Friday, or 9:00 am to 5:00 pm, Saturdays and Sundays)? (Reason: Cost)	CCHS
	ACC_67	In the past 12 months, did you ever experience any difficulties getting the immediate care needed for a minor health problem for yourself or a family member that you required during the middle of the night? (Reason: Cost}	CCHS
	PCU_56	What are the reasons that you have not had a check-up in the past 3 years? (Reason: Cost)	CCHS
	RAH_09	In the past 12 months, how often did you find it difficult to get healthcare services because you could not travel to the healthcare facility? What types of problems did you experience? (Reason: I can’t afford the cost of travel/I can’t afford to take time off work)	BCPCHC
	RAH_18	In the past 12 months, did you ever experience any travel-related or other difficulties when trying to see a medical specialist? What types of problems did you experience? (Reason: I can’t afford the cost of travel/I can’t afford to take time off work)	BCPCHC
	RAH_09	In the past 12 months, how often did you find it difficult to get healthcare services because you could not travel to the healthcare facility? What type of problems did you experience? (Reason: I can’t afford the cost of travel, I can’t afford to take time off work, I can’t afford the cost of parking)	BCPCHC
	GBC_03	In the past 12 months, what type of difficulties did you experience getting any healthcare services from your family doctor or general practitioner? (Reason: Cost)	BCPCHC
	GBC_05	In the past 12 months, what type of difficulties did you experience getting specific care for your chronic condition? (Reason: Cost)	BCPCHC
	WTM_06	In the past 12 months, did you require a visit to a medical specialist for a diagnosis or a consultation for a new illness or condition? Thinking about this visit, did you experience any difficulties seeing the specialist? What type of difficulty did you experience? (Reason: Cost)	CCHS
	BPC_16 RC_01C	What are the reasons you have not had your blood pressure taken in the past 2 years? (Reason: Cost)	CCHS BCPCHC
	RC_02B	What are the reasons you have not had your cholesterol measurement taken in the past year? (Reason: Cost)	BCPCHC
	RC_03B	What are the reasons you have not had your blood sugar taken in the past year? (Reason: Cost)	BCPCHC
	ACC_32	In the past 12 months, did you ever experience any difficulties getting the tests you needed? (Reason: Cost)	CCHS
	WTM_37	In the past 12 months, did you require a diagnostic test? Did you experience any difficulties getting this test? What type of difficulty did you experience (Reason: Cost)	CCHS
	EYX_142	When did you last have an eye examination? What are the reasons that you have not had an eye examination in the past 2 years? (Reason: Cost)	CCHS
	DEN_132	When was the last time that you went to a dentist? What are the reasons that you have not been to a dentist in the past 3 years? (Reason: Cost)	CCHS
Medication	PDE_02	In the past 12 months, did you decide not to fill a new prescription for medication because of the cost? (Yes)	PDERR
	PDE_03	In the past 12 months, did you decide not to renew a prescription for medication because of the cost? (Yes)	PDERR
	PDE_04	In the past 12 months, because of the cost, did you do anything to make a prescription medication last longer? (Yes)	PDERR
	MEH_05	What are the reasons that you are not currently taking any prescription medications for your high blood pressure? (Reason: Cost)	SLCDC
	MU_05	Over the past 12 months, have you ever stopped taking one or more of your drugs as prescribed for a week or more? What are the reasons that you did not take your medication as prescribed? (Reason: Cost)	BCPCHC
	INS_03	Do you currently have insurance that covers all or part of the cost of prescription medications? Why not? (Reason: I can’t afford coverage)	BCPCHC
Home care	HMC_15 CR1_04	During the past 12 months, was there ever a time when you felt that you needed home care services but you didn’t receive them? (Reason: Cost)	CCHS
Unmet need	EBC_01	In the past 12 months, how often have you had difficulty paying for services, equipment or medications you need to help you manage your chronic condition? (Always, often, sometimes)	BCPCHC
	HCU_06 UCN_01	During the past 12 months, was there ever a time when you felt that you needed healthcare, but you didn’t receive it? (Reason: Cost)	CCHS
Healthful food	FIN_01	In the past 12 months, how often did you or anyone else in your household worry that there would not be enough to eat because of a lack of money? (Often, sometimes)	CCHS
	FIN_02	In the past 12 months, how often did you or anyone else in your household not have enough food to eat because of a lack of money? (Often, sometimes)	CCHS
	FIN_03	In the past 12 months, how often did you or anyone else in your household not eat the quality or variety of foods that you wanted to eat because of a lack of money? (Often, sometimes)	CCHS
	FSC_10	Which of the following statements best describes the food eaten in your household in the past 12 months? (Sometimes did not have enough, often did not have enough)	CCHS
	FSC_20	You and other household members worried that food would run out before you got money to buy more. Was that often true, sometimes true or never true in the past 12 months? (Often true, sometimes true)	CCHS
	FSC_30	The food that you and other household members bought just didn’t last, and there wasn’t any money to get more. Was that often true, sometimes true or never true in the past 12 months? (Often true, sometimes true)	CCHS
	FSC_50	You or other adults in your household relied on only a few kinds of low-cost food to feed the children because you were running out of money to buy food. Was that often true, sometimes true or never true in the past 12 months? (Often true, sometimes true)	CCHS
	FSC_80	In the past 12 months, did you or other adults in your household ever cut the size of your meals or skip meals because there wasn’t enough money for food? (Yes)	CCHS
	FSC_90	In the past 12 months, did you (personally) ever eat less than you felt you should because there wasn’t enough money to buy food? (Yes)	CCHS
	FSC_100	In the past 12 months, were you (personally) ever hungry but didn’t eat because you couldn’t afford enough food? (Yes)	CCHS
	FSC_110	In the past 12 months, did you (personally) lose weight because you didn’t have enough money for food? (Yes)	CCHS
	FSC_120	In the past 12 months, did you or other adults in your household ever not eat for a whole day because there wasn’t enough money for food? (Yes)	CCHS
	SMH_04 SMH_02	What are the reasons you are not limiting your daily salt intake? (Reason: Cost)	BCPCHC SLCDC
	SMH_08 SMH_04	What are the reasons you are not choosing these types of foods (that is, fruits and vegetables, fish or lean meats, foods high in fibre or foods low in fat)? (Reason: Cost)	BCPCHC SLCDC1
	SMD_02	What are the reasons you do not change the type or amount of food you eat to help control your diabetes? (Reason: Cost)	SLCDC2
Health behaviour modification	SMH_08 SMD_04 SMH_10	What are the reasons that you are not exercising or participating in physical activities? (Reason: Cost)	SLCDC1 SLCDC2 BCPCHC
	PA2_13	What prevented you from doing more physical activities? (Reason: Cost)	CCHS
	SMH_10 SMD_06 SMH_21	What are the reasons that you are not trying to control your weight or lose weight? (Reason: Cost)	SLCDC1 SLCDC2 BCPCHC
	SMH_17	What are the reasons that you are not trying to quit smoking or cut down on smoking (Reason: Cost of smoking cessation products)	BCPCHC

*CCHS* Canadian Community Health Survey

*BCPCHC* Barriers to Care for Persons with Chronic Health Conditions (2012)

*SLCDC1* Survey on Living with Chronic Diseases in Canada (hypertension component) (2009)

*SLCDC2* Survey on Living with Chronic Diseases in Canada (diabetes component) (2011)

*PDERR* Rapid Response Module on Prescription Drug Expenditures (2007)

## Appendix 2

Table 5 lists the associated administrative data codes.

**Table 5:** Definition of primary outcome

Description	ICD-9 code	ICD-10 code	CCP code
Diseases of the cardiovascular system	390.x-459.x	Ixx	
Diabetes mellitus	250.X	E10-E14	
	251		
Pulmonary edema	518.4	J81	
Coronary artery bypass grafting	36.10-36.19	1.IJ.76	48.11-48.19
Percutaneous coronary intervention	36.01-36.02	1.IJ.50	48.02-48.03
	36.05	1.IJ.57.GQ	
		1.IJ.54	
		1.IJ.76	

## Abbreviations

Avg: Average
CCHS: Canadian Community Health Survey
CI: Confidence interval
CMG: Case-Mix Grouper
GLM: Generalized linear model
IRR: Incidence rate ratio
OLS: Ordinary least squares
